# Visualization of protein interactions in living *Drosophila *embryos by the bimolecular fluorescence complementation assay

**DOI:** 10.1186/1741-7007-9-5

**Published:** 2011-01-28

**Authors:** Bruno Hudry, Séverine Viala, Yacine Graba, Samir Merabet

**Affiliations:** 1 Institut de Biologie du Développement de Marseille Luminy, IBDML, UMR 6216, CNRS, Université de la méditerranée, Parc Scientifique de Luminy, Case 907, 13288, Marseille Cedex 09, France

## Abstract

**Background:**

Protein interactions control the regulatory networks underlying developmental processes. The understanding of developmental complexity will, therefore, require the characterization of protein interactions within their proper environment. The bimolecular fluorescence complementation (BiFC) technology offers this possibility as it enables the direct visualization of protein interactions in living cells. However, its potential has rarely been applied in embryos of animal model organisms and was only performed under transient protein expression levels.

**Results:**

Using a Hox protein partnership as a test case, we investigated the suitability of BiFC for the study of protein interactions in the living *Drosophila *embryo. Importantly, all BiFC parameters were established with constructs that were stably expressed under the control of endogenous promoters. Under these physiological conditions, we showed that BiFC is specific and sensitive enough to analyse dynamic protein interactions. We next used BiFC in a candidate interaction screen, which led to the identification of several Hox protein partners.

**Conclusion:**

Our results establish the general suitability of BiFC for revealing and studying protein interactions in their physiological context during the rapid course of *Drosophila *embryonic development.

## Background

Since its cloning in 1992 [1], the green fluorescent protein (GFP) from the jellyfish *Aequorea victoria *has become a potent tool in biology. GFP has been manipulated and modified and a set of derived fluorescent proteins showing different spectral properties and stabilities are now available. GFP proteins can tolerate peptide insertions without perturbing its fluorescence characteristics [2,3]. This led to the finding that split GFP fragments can reconstitute a functional fluorescent protein when fused to interacting peptides [4]. First applied in *Escherichia coli *[4] and Hela cells [5], the visualization of protein interactions *in vivo *was definitively validated by the work of Hu *et al*., who investigated the interactions between transcription factors in mammalian cells [6].

This protein interaction assay, termed BiFC for bimolecular fluorescence complementation, uses a number of protein variants derived either from the GFP [yellow fluorescent protein (YFP), Venus and Cerulean] [7] or from other fluorescent proteins (mRFP1 [8], mCherry [9] and Dronpa [10]). Protein interactions are visualized using a standard epifluorescence microscope and the analysis does not require complex data processing. Combining peptides of different fluorescent proteins for complementation (termed multicolour BiFC) expanded the number of protein interactions that could be simultaneously visualized [7,11,12].

BiFC is by now a widely used assay for testing the protein interaction status in cultured cells [13] and plants [14]. In contrast, only a handful studies have used the technology for the visualization of protein interactions in animal model organisms. In vertebrates, BiFC has been performed in *Xenopus laevis *and *Danio rerio *to visualize the nuclear translocation of a Smad2/Smad4 complex in embryonic explants [15,16] or embryos [17], respectively. In invertebrates, BiFC was used to reveal interactions between proteins of gap junctions [18] or between leucine zipper polypeptides [19] in *Caenorabditis elegans *and between odorant receptors [20], actin nucleation proteins [21] or transcription factors [22] in *Drosophila melanogaster*. Importantly, these latter studies were only undertaken in adults.

The absence of BiFC applications in the embryos of these model organisms might appear somewhat surprising, given that they have been extensively used to address questions relating to mechanisms underlying developmental control, and that protein interactions are central to the developmental processes. Among the reasons that may explain this are technical constraints, such as the short time window characterizing worm and fly embryogenesis, and the sensitivity of the technique when fusion proteins are expressed at the same level as the endogenous proteins of interest, a prerequisite for drawing physiologically relevant conclusions.

In this work, we question the suitability of the BiFC technology in conditions where proteins are expressed at physiological levels in the *Drosophila *embryo, using the partnership between the Hox protein AbdominalA (AbdA) and the PBC class Extradenticle (Exd) protein as a paradigm. Hox proteins are present in all bilaterians and play major roles during embryonic development by controlling diversified morphogenesis along the anteroposterior axis [23]. Hox and PBC proteins are both homeodomain (HD) containing transcription factors and have been shown to work in many instances by assembling regulatory complexes on *cis*-regulatory sequences of target genes [24].

Here, we establish experimental parameters that make BiFC compatible with both the physiological levels of protein expression and the short time window characterizing *Drosophila *embryogenesis. Our work demonstrates that BiFC is a specific and sensitive method which allows the visualization of spatial interaction dynamics between AbdA and Exd. The technology was further used for assessing the *in vivo *interaction status between AbdA and several candidate partners, including non-HD containing transcription factors. Results indicate that the methodology is generally suited for the study of interactions between different types of transcription factors.

## Methods

### Fusion protein constructs and transgenic lines

The DNA fragments coding for the N-terminal (VN: 1-173) and C-terminal (VC: 155-238) moieties of Venus were generated by polymerase chain reaction (PCR) and cloned into EcoRI-XhoI or XhoI-XbaI restriction sites of the pUAST [25] and pUASTattB [26] vectors for 5' and 3' fusions, respectively. The DNA inserts to obtain fusions downstream the Venus fragment were cloned into XhoI-XbaI sites, while inserts to generate fusions upstream the Venus fragment were cloned into EcoRI-XhoI sites. Inserts coding for Hox, Exd and candidate partner proteins were generated by PCR from full length complimentary DNAs (cDNA). Exd fusion proteins were also HA-tagged. In all constructs, a linker of five amino acids was added to separate the Venus fragment from the protein of interest. Primers used are available upon request.

Cerulean N-terminal (CN: 1-173) and C-terminal (CC:155-238) coding fragments [7] were cloned into EcoRI-XhoI sites of pUAST for generating 5' fusions with AbdA and Exd proteins. mCherry [9] N-terminal (mCN: 1-159) and C-terminal (mCC:159-237) coding fragments were cloned into EcoRI and XhoI sites of pUASTattB, respectively, for generating 5' fusions with AbdA and Exd.

All constructs were sequence-verified before fly transformation and transgenic lines were established either by the ΦC-31 integrase [26] Ultrabithorax (Ubx), AbdA and Exd variants, as well as fusions with split mCherry fragments] or by classical P-element (nlsVN, VC, HthVN, VCHth, VNTFIIbeta, BIP2VN, TshVN, VNBin, as well as AbdA and Exd constructs with split fragments of Cerulean) mediated germ line transformation.

### Fly stocks

The *abdAGal4 *line was generated by replacing a *P-lacZ *insertion (line HC7JA1 [27]) by a *P-Gal4 *element [28]. Other Gal4 drivers used are: *armadillo(arm)-Gal4*, *paired(prd)-Gal4*, *engrailed (en)-Gal4*, *24B-Gal4*, *breathless (btl)-Gal4 *and *Ubx-Gal4*^*M1 *^[28] drivers. *UAS-abdA*, UAS-Bip2, UAS-β-Galactosidase and *exd *^*XP11 *^lines were obtained from the Bloomington Stock Centre (IN, USA); the UAS-Tsh line was kindly provided by S Kerridge.

### Cuticle preparations and immunostaining

Embryo collections, cuticle preparations and immunodetections were performed according to standard procedures [29,30]. The antibodies used were: chicken anti-GFP (Promega, WI, USA; 1/500), mouse anti-ß-galactosidase (Molecular Probe, Invitrogen, CA, USA; 1/500), rat anti-HA (Molecular Probe, 1/500), rabbit anti-AbdA (Dm.Abd-A.1, 1/1000), mouse anti-Ubx (FP3.38, 1/100).

### *In vivo *quantification of protein expression levels

Experimental conditions allowing physiological levels of protein expression were established by two steps. First, the VC-AbdA construct was expressed with the *arm-Gal4 *driver at different temperatures. The anti-AbdA fluorescent immunostaining was compared between the A2 segment of wild-type embryos and the T2 segment of embryos ectopically expressing VC-AbdA. Pictures of at least 10 different embryos were taken with a LSM510 Zeiss confocal microscope under fixed parameters of acquisition and average levels of pixel intensities were determined with the ImageJ software. The ratio between the experimentally induced AbdA protein levels in T2 and the wild-type AbdA protein levels in A2 was determined, allowing the selection of the temperature for which the induced AbdA expression with *arm-Gal4 *was close to the endogenous AbdA expression levels found normally in A2.

Secondly, we established experimental conditions for using the *abdAGal4 *driver. This was performed with the VC-AbdA construct: intensities of the GFP fluorescent immunostaining were measured and compared to embryos expressing VC-AbdA with the *arm-Gal4 *driver in order to adjust the physiological conditions of expression. In our conditions, *abdA-Gal4 *is 20% more expressed than *arm-Gal4*. Since *arm-Gal4 *led to 80% of the AbdA endogenous expression levels in the A2 segment, we concluded that our experimental conditions with *abdA-Gal4 *are close to physiological levels of expression.

### BiFC visualization in living embryos

Fly crosses for BiFC analyses were set up at the defined temperature over night. After the removal of the flies, the embryos were kept at 4°C for 28 h before live imaging. In order to visualize the complementation between split Venus fragments, living embryos were dechorionated and mounted in the halocarbon oil 10S (commercialized by VWR, Pennsylvania, USA). In order to quantify the BiFC signals, unsaturated images of ectodermal fluorescence were taken in embryos of the desired stage (with a minimum of 10 embryos by condition) using a LSM510 confocal microscope (Zeiss, Jena, Germany). For Venus fluorescence, filters were adjusted at 500 nm for excitation and 535 nm for emission. Identical parameters of acquisition were applied between the different genotypes. The number and intensity of the all pixels (for each embryo) were measured using the histogram function of the ImageJ Software. The quantification of fluorescence complementation was shown for each condition by boxplot representation using R-Software. Boxplot depicts: the smallest value, lower quartile, median (green line), upper quartile and largest value for each condition. Black points correspond to individual measures. Cerulean and mCherry BiFC signals were taken after identical maturation times of 28 h with the AxioImagerZ1 microscope (Zeiss), using specific filters for excitation and emission wavelengths (440/475 nm for Cerulean, 580/610 nm for mCherry).

### Test of the lethality induced by incubation times at 4°C

A fixed number of 100 embryos were collected over a 1h period at room temperature and left to develop for 4h, which allows having a high proportion of stage 9/10 embryos. Embryos were next placed at 4°C for different times before being returned to resume and complete development at room temperature. The embryonic lethality rate was deduced by counting the number of hatching embryos that gave rise to first instar larvae. For each incubation time the experiments were repeated twice and the results were stably reproduced.

### Protein expression and electrophoretic mobility shift assays (EMSAs)

The fusion constructs were subcloned in the PcDNA3 vector and sequence-verified. Proteins were produced with the TNT T7-coupled *in vitro *transcription/translation system (Promega). Production yields of AbdA and Exd fusion proteins were estimated by ^35^S-methionine labelling. The amount of Hox proteins used in the band shift assays is indicated in the figure legends. EMSAs were performed as described previously [31]. We used double strands radiolabelled DllR^con ^5'-TATTTGGGCCATAAATCATTCCCGCGGACAGTT-3' [32] and PRS 5'-TTAGCGCGGGCGCATCAATCAATTTTCG-3' probes [33]. The rabbit anti-AbdA and mouse anti-Exd antibodies were used at a 1/100 dilution for the 'supershift' experiments.

## Results

### Influence of the fusion topology on AbdA-Exd complex formation *in vitro*

**Figure 1:**
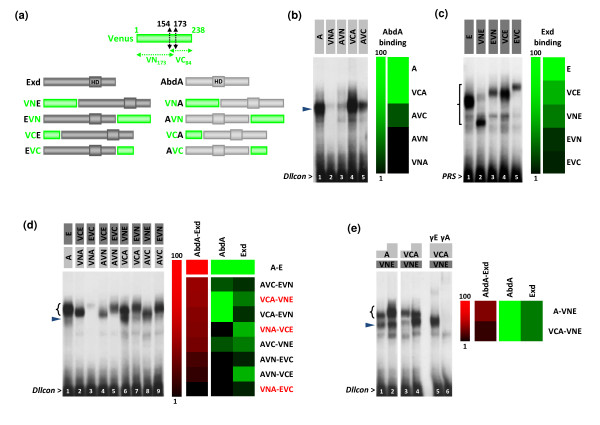
**Influence of the identity and position of fusions on the AbdA-Exd (abdominalA-extradenticle) complex assembly *in vitro***. (a) A schematic representation of the fusion proteins realized between Exd (dark grey), AbdA (light grey) and split Venus fragments (VN, green). The homeodomain (HD) of AbdA and Exd is also indicated. (b) Monomere DNA binding activities of AbdA fusion proteins (black arrowhead) on the *Distalless *consensus probe (*Dllcon*). (c) Monomere DNA binding activities of Exd fusion proteins (bracket) on the Pbx1 recognition sequence (*PRS*) probe. Note that migrations do not necessarily correspond to the size of the protein since electrophoretic mobility shift assays (EMSAs) are performed in non-denaturated conditions. Diagrams on the right classify the fusion proteins as a gradient of their DNA binding affinities. This representation was obtained from the quantification of each band and values were clustered with the *MultiExperiment Viewer *(MeV) software. (d) EMSA with all combinations of AbdA and Exd fusion proteins on the *Dllcon *probe, as indicated above the gel. The diagram on the right was obtained as in (b) and (c). Combinations of fusion proteins used for the *in vivo *bimolecular fluorescence complementation (BiFC) analysis are highlighted in red. The black arrowhead indicates the monomer binding of AbdA proteins. (e) Comparison of the efficiency of complex formation between AbdA or VC-AbdA (VCA) and VN-Exd (VNE). The bracket indicates dimers and black arrowhead AbdA monomers, as confirmed by supershifts with the anti-Exd (lane 5) and anti-AbdA (lane 6) antibodies. The diagram on the right illustrates the level of complex formation in comparison to monomer DNA binding affinities, as in (d). All EMSAs were performed with identical amounts of Exd (40 ng) and AbdA (20 ng) proteins, except in (e) where 20 ng and 40 ng of AbdA have been used (as illustrated by white-grey boxes above the gel).

The identity (corresponding to the VN and VC fragments: Figure 1a) and the position (at the N-terminus or C-terminus of the protein) of the split fragments of a fluorescent protein, collectively referred to as topology, may influence the function and the interacting potential of the fusion protein. Although widely recognized as being critical for BiFC, the influence of these two parameters has never been systematically addressed [13]. We therefore investigated whether an *in vitro *approach based on EMSAs could help in predicting the best appropriate choice of fusion proteins for *in vivo *analyses.

Indeed, EMSAs allow one to measure both DNA binding and protein interactions. Each of the split fragments of the Venus fluorescent protein was fused to the N-terminus or the C-terminus of AbdA and Exd (Figure 1a). Importantly, all fusion proteins were constructed with an identical five amino acids linker region of about 20 angstroms long (see Methods). Although this short linker length could limit the efficiency of fluorescence complementation [13], it was preferred over longer linkers in order to avoid artificial complementation in the absence of protein interactions. We considered this to be of particular importance for transcription factors, since the proximity of binding sites on the DNA target, or chromatin looping, could bring proteins in close proximity without implying direct interactions between them. EMSAs were performed with two different probes, both of which corresponded to consensus DNA target sites. Consensus binding sites were preferred over physiological sites since they probably better reflect the general DNA binding properties of a given transcription factor. The first probe, called *Dllcon *(for *Distalless *consensus), corresponds to a consensus Hox-Exd DNA binding site for the AbdA monomer and the AbdA-Exd heterodimer [34]. The second one, called *PRS *(for Pbx1 recognition sequence), corresponds to a monomer binding site for PBC proteins *in vitro *[33]. Together, these probes allow us to measure monomer DNA-binding activities and heterodimer formation separately, enabling conclusions to be drawn on the AbdA/Exd interaction potential.

EMSAs with single proteins show that the fusion topology is not neutral for DNA binding. In AbdA, the VN (Venus) fragment strongly inhibits monomer DNA binding while the VC fragment has minor effects (Figure 1b, compare lanes 2-3 to 4-5 and the quantitative diagram). In Exd, it is the position of the fusion that is important, with fusions at the C-terminus affecting the DNA binding activity more strongly than fusions at the N-terminus (Figure 1c, compare lanes 3 and 5 to lanes 2 and 4, and the quantitative diagram). For heterodimer formation, EMSAs with all eight possible combinations between AbdA and Exd fusion proteins showed that the protein topologies providing the highest levels of monomer binding did not necessarily yield to highest levels of heterodimeric complexes (Figure 1d). For example, the best combination for heterodimeric formation corresponds to AbdA-VC/Exd-VN, although these two fusion proteins did not show the strongest monomer DNA binding activities (compare the red and green quantitative diagrams in Figure 1d). This observation suggests that fusion topologies also affect the AbdA-Exd interaction. In order to confirm this point, we compared the efficiency of complex formation between proteins with equivalent monomer binding affinities such as the AbdA/VN-Exd and VC-AbdA/VN-Exd combinations (green quantitative diagrams in Figure 1e). Results show that the VC-AbdA fusion protein formed fewer complexes with VN-Exd than did the unfused AbdA (Figure 1e, compare lanes 3-4 to 1-2). As both AbdA proteins have equivalent DNA binding properties on *Dllcon *(Figure 1b, lanes 1 and 4), we conclude that the fusion of the VC fragment at the N-terminus of AbdA impaired protein interactions with Exd.

Our extensive *in vitro *analysis highlights the fact that the topology of the fusions can drastically affect complex formation between AbdA and Exd. Although this was expected in general, it was not possible to predict which topology would actually be the most neutral to protein activity. Therefore, EMSA may constitute a valuable rapid assay for choosing the best match of fusion proteins for BiFC analysis in the case of DNA binding transcription factors.

### Expressing fusion proteins at endogenous levels

**Figure 2:**
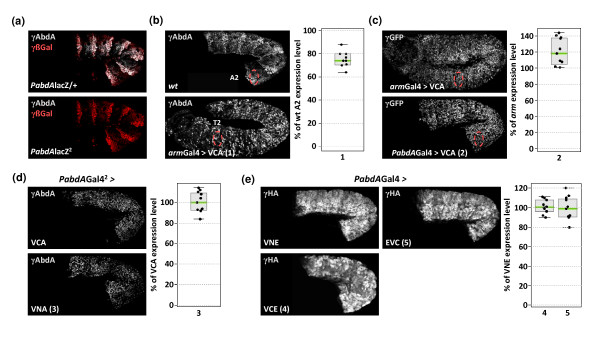
**Establishing physiological levels of fusion protein expression**. (a) A P-element insertion in the *bithorax *locus abolishes *abdA *expression and reproduces the expression profile of *abdA*. Compared to heterozygous embryos (upper panel), AbdA (abdominalA) expression (grey) is absent in embryos homozygous for the P insertion (named *HC7JA1 *[27]) that contains the β-galactosidase (β-Gal, red) reporter protein (bottom panel). (b) Establishing physiological levels of VCA expression with the *armadillo (arm)-Gal4 *driver. The average level of VCA expression at 29°C is quantified in the T2 thoracic segment and compared to the level of endogenous AbdA in the A2 segment of a wild type embryo (red-dotted circles). Fluorescent immunostainings were similarly performed with an anti-AbdA antibody (grey). Graph on the right is a boxplot representation of the statistical quantification of the surface and intensity of the fluorescent AbdA immunostaining (see also Methods). (c) Establishing physiological levels of expression with the *abdA-Gal4 *driver. Quantifications were measured with an anti-green fluorescent protein that recognises the VC fragment of VCA. Fluorescent immunostainings (grey) were performed in embryos expressing VCA either with *arm-Gal4 *or *PabdA-Gal4 *at 29°C. (d) The VC-AbdA (VCA) and VN-AbdA (VNA) fusion proteins are expressed at similar levels. Stage 10 embryos homozygous for the *PabdA-Gal4 *driver (symbolized by the exponent) and carrying one copy of VCA or VNA are stained with anti-AbdA antibody (grey). In these embryos, endogenous AbdA is absent, revealing the expression level of AbdA fusion proteins only. (e) The VN-extradenticle (Exd; VNE), VC-Exd (VCE) and Exd-VC (EVC) fusion proteins are expressed at similar levels. Exd fusion proteins are HA-tagged and were expressed with the *abdA-Gal4 *driver, as indicated. Graph on the right shows the level of fluorescent immunostaining of EVC (4) and VCE (5) when compared to levels of VNE. Fluorescent immunostaining (gray) against the HA tag was quantified as previously.

In order to investigate whether EMSAs could predict the influence of fusion topologies on both *in vivo *functions and BiFC efficiency, we selected combinations which showed weak (VC-AbdA/VN-Exd), medium (VN-AbdA-VC-Exd) or strong (VN-AbdA/Exd-VC) impairments for complex assembly *in vitro *(these combinations are highlighted in red in Figure 1d). Transgenic lines allowing expression of the corresponding AbdA and Exd fusion proteins under the UAS/Gal4 system were established. Importantly, all UAS constructs were inserted at the same genomic locus through the use of the phiC31-integrase system [26], ensuring comparable expression levels in the embryo. In order to be close to physiological conditions of expression, we generated an *abdA-Gal4 *driver by replacing a *P- lacZ *insertion in the *abdA *cis-regulatory sequences [28] by a *P-Gal4 *element (Figure 2a). This *P-Gal4 *insertion corresponds to an *abdA *null mutation (Figure 2a), which results in the absence of competitive endogenous AbdA proteins. We then selected experimental conditions where the *abdA-Gal4 *driver generated expression levels similar to endogenous *abdA *(see Methods). This was performed by quantifying the activity of the *armadillo (arm)-Gal4 *driver (Figure 2b), which then served as a reference value for the selection of proper expression levels of the VC-AbdA (Figure 2c) and VN-AbdA (Figure 2d) fusion proteins with the *abdA-Gal4 *driver. Exd fusion proteins were expressed with the same *abdA-Gal4 *driver (Figure 2e), which did not recapitulate the ubiquitous expression pattern of the endogenous Exd [35]. However, this was of minor consequence since the nuclear distribution of Exd, and hence its function, is dependent of the presence of homothorax (Hth), another HD-containing protein ([36] and below). Consequently, ectopic expression of Exd reproduces the endogenous nuclear distribution in the embryo [35], showing that Exd fusion proteins are not localized in all nuclei of *abdA*-expressing cells (Figure 2e). In conclusion, our genetic tools allow the expression of AbdA and Exd fusion proteins at levels and in places close to physiological conditions.

### Influence of the fusion topology on AbdA and Exd functions

**Figure 3:**
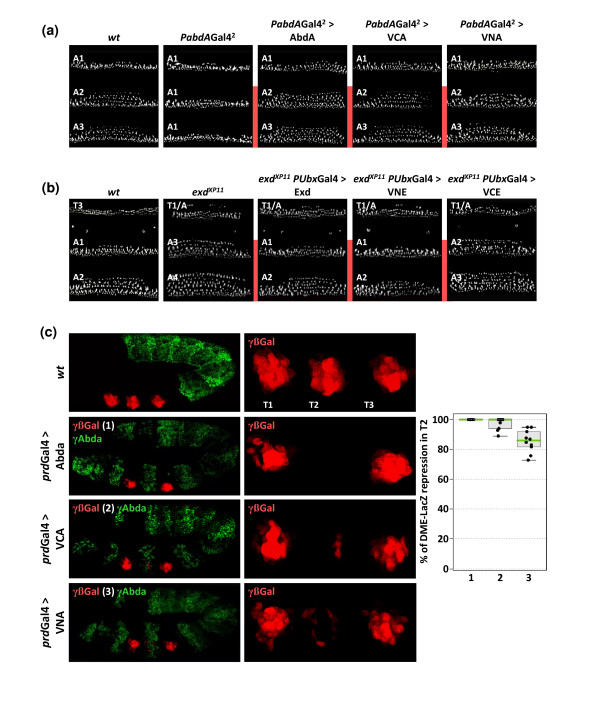
**Influence of fusion topologies on abdominalA (AbdA) and extradenticle (Exd) functions *in vivo***. (a) Cuticle phenotypes of *abdA *mutants (homozygous for *PabdA-Gal4*) and rescue activities by AbdA fusion proteins. Mutant larvae are characterized by the loss of denticle rows in A2-A7 segments, presenting a thinner A1-like organization. Enlargements are focused on the A1-A3 segments. Expression of AbdA or VCA in this mutant context restores the A2-like shape of denticle rows. Expression of VNA is less efficient in the rescue, leading to an intermediary A1/A2 organization of denticle belts. Red lines indicate the anterior expression boundary of the *abdA-Gal4 *driver. (b) Rescue of *exd *cuticle phenotypes by Exd fusion proteins. In zygotic *exd*^*XP11 *^mutant embryos, the T3 segment acquires a mix T1/abdominal identity, while the A1 and A2 segments resemble to more posterior abdominal segments (respectively to A3-like and A4-like segments). The rescue efficiency of Exd fusion proteins was measured in abdominal segments by using the *Ultrabithorax(Ubx)-Gal4 *driver ([28] and Additional File 1). Abdominal phenotypes were rescued by Exd or VNE and VCE (not shown) fusion proteins. EVC led to an intermediary rescue, with A1 and A2 segments acquiring respectively an A2-like and A3-like morphology. (c) Regulatory effects of AbdA fusion proteins on the *Distalless *(*Dll*) enhancer *DME *[26]. β-Galactosidase (β-Gal) immunostaining (red) reveals the expression of a *lacZ *reporter gene that is under the control of *DME cis*-regulatory sequences. Ectopic expression of AbdA (green) in the thoracic T2 segment with the *paired (prd)-Gal4 *driver led to complete repression of the β-Gal. VCA, and to a lesser extend VNA, are also able to repress *DME*. Graph on the right shows the statistical quantification of the repression of the β-Gal by AbdA (1), VCA (2) and VNA (3), as deduced from the level of the red fluorescent signal in T2.

As we observed that Venus fusions can impact on AbdA and Exd DNA binding and heterodimer formation, we first investigated their activity *in vivo*. We used the rescue of the altered segmental morphology of zygotic *abdA *and *exd *mutants as an assay for *in vivo *protein activity [37]. The measurement of the efficiency of the rescue obtained with AbdA fusion proteins was performed in embryos homozygous for the *abdA-Gal4 *driver, which resemble *abdA *null mutants (Figure 3a). The activity of Exd fusion proteins was assayed with an *Ubx-Gal4 *driver [28], allowing the rescue in more segments than by using *abdA-Gal4 *(Figure 3b). As described for the *abdA-Gal4 *driver, the *Ubx-Gal4 *driver was used at experimental conditions ensuring physiological rates of protein expression (see Additional File 1). In all cases, the efficiency of the rescue can only be determined in the expression domain of the corresponding drivers (highlighted by a red line in Figure 3a and 3b). We observed that fusions which weaken monomer binding (VN-AbdA and Exd-VC) partially rescue the mutant phenotypes, while fusions that do not affect monomer DNA binding (VC-AbdA and VN-Exd) led to a complete rescue of the phenotype, comparable to that obtained with the wild-type AbdA and Exd proteins (Figure 3a and 3b). In addition to the cuticle phenotype, the activity of AbdA fusion proteins was also tested on the *Distalless *(*Dll*) target gene. Through its *DME *enhancer, this gene is normally repressed by AbdA in the abdomen [34]. Consequently, ectopic expression of AbdA with the *paired-Gal4 *driver leads to complete repression of *DME *in the T2 thoracic segment (Figure 3c). The VC-AbdA fusion protein was also able to strongly repress *DME*, while the VN-AbdA behaved as a less potent repressor (Figure 3c).

In conclusion, fusions with Venus fragments can affect activity *in vivo*, potentially leading to hypoactive proteins, as observed for VN-AbdA and Exd-VC fusion proteins. Interestingly, however, the *in vivo *activity of the proteins correlates well with their *in vitro *activity assessed by EMSA.

### Establishing experimental parameters in order to visualize BiFC from the AbdA/Exd complex assembly in the *Drosophila *embryo

**Figure 4:**
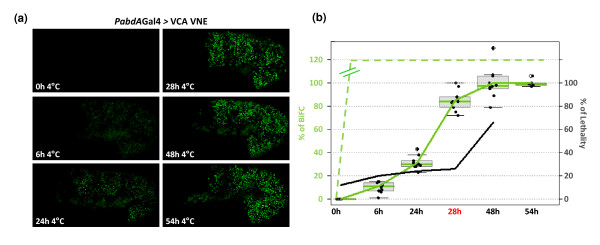
**Influence of incubation times at 4°C on the level of bimolecular fluorescence complementation (BiFC) signals and embryonic lethality**. (a) Influence of various incubation times on the level of BiFC signals. BiFC resulted from the expression of the VCA and VNE fusion proteins with the *abdA-Gal4 *driver. Confocal images of stage 10 embryos were taken under same parameters of acquisition after various periods of incubation at 4°C, as indicated. (b) Statistical representation of the effect of incubation times on the embryonic lethality (black curve) and on BiFC levels resulting from the VCA/VNE assembly (green curve) or from VN/VC interactions (dotted-green curve, not quantified with regard to VCA/VNE, as symbolised by double lines). An incubation time of 28 h (highlighted in red) was considered as best appropriate for visualizing BiFC with fusion proteins (with a corresponding low rate of lethality and high level of fluorescence). This time was systematically applied for BiFC observations described in the following figures. See also Material and Methods.

The fluorescence intensity emitted by BiFC complexes *in vivo *is generally less than 10% of that produced by the corresponding full length fluorescent proteins. This may be due to the presence of multiple interacting partners which means that only a subset of fusion proteins will associate with each other in living cells [38]. Moreover, in fluorescence complementation assays, fluorophore maturation causes a delay between fusion proteins interaction and the appearance of fluorescence. These two features may compromise BiFC applications to the *Drosophila *embryo, which is characterized by a fast development and a background autofluorescence. In order to circumvent these limitations, embryos at the desired developmental stages were placed at 4°C for different periods of time before live imaging. That is the temperature which stops embryonic development, but which permits fluorescence maturation as long as the split Venus fragments are associated. Thus, the temperature shift allows the bypassing of the normal time delay for BiFC detection. Assays were performed with the VC-AbdA and VN-Exd fusion proteins, which showed *in vitro *and *in vivo *activities close to wild-type parental proteins. We found that the minimal incubation time required for visualizing fluorescent signals is 6 h (Figure 4a). Under these conditions, BiFC resulting from the VC-AbdA/VN-Exd complex assembly in the *Drosophila *embryo was barely visible. By keeping embryos at 4°C for longer periods, BiFC signals were significantly increased (Figure 4a). In particular, incubation times comprised between 24 h and 28 h led to the production of BiFC signals that peaked to 30% and 85%, respectively, of the maximum level of fluorescence intensity (green curve in Figure 4b). The fluorescence intensity reached its maximum after 48 h and remained constant till 54 h (Figure 4b). These results establish that keeping embryos at 4°C allows the visualization of protein interactions that would otherwise not be detected due to the short time window of *Drosophila *embryonic development.

As BiFC can only be visualized after a period of incubation at 4°C, we also tested the impact of this experimental procedure on the *Drosophila *embryo viability. This was performed by counting the percentage of embryos that gave rise to first instar larvae when development was resumed at room temperature after incubation at 4°C (see also Methods). As a control, we found that embryos have a normal rate of lethality around 12% when development occurred at room temperature only (black curve in Figure 4b). This rate did not importantly increase when embryos were kept at 4°C for a period ranging from 6 h to 28 h, with an average lethality of 22% (Figure 4b). Embryonic development was more severely affected by longer times of incubation, with 66% of lethality at 48 h (Figure 4b). Of note, we observed that all embryos processed for BiFC after an incubation time of 28 h developed normally until stage 14 (as illustrated in the movie of Additional File 2) which, given the rate of non-hatching embryos, was not expected. We suggest that the lethality might occur at very early or late embryonic stages, allowing BiFC analyses only in developing embryos.

From these experiments, we concluded that the best incubation time at 4°C to observe BiFC from the VC-AbdA/VN-Exd complex assembly was 28 h. Under these conditions, BiFC signals were strong enough and embryonic viability was not significantly affected. This incubation time was systematically applied for further BiFC assays.

### Influence of the fusion topology on BiFC signals

**Figure 5:**
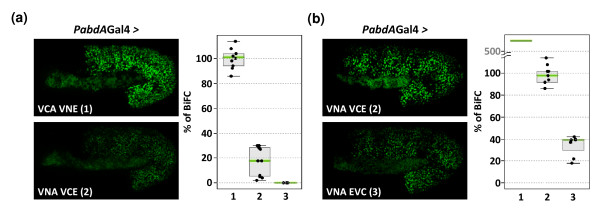
**Influence of fusion topologies on bimolecular fluorescence complementation (BiFC) signals resulting from abdoninalA/exradenticle (AbdA/Exd) complex assembly**. (a) The brightness of fluorescence resulting from BiFC varies depending of the combination of AbdA and Exd fusion proteins: VCA/VNE (1) produced strong BiFC, while VNA/VCE (2) and VNA/EVC (3) complexes produced weak and no BiFC under same parameters of confocal acquisition, respectively. (b) Compared to (a), the laser power is increased to its maximum in order to visualize BiFC resulting from the VNA/EVC complex assembly. Consequently, BiFC signals resulting from VCA/VNE are saturated and the corresponding quantification can only be estimated under such parameters of acquisition. See also Additional File 3.

In order to investigate whether BiFC efficiency could be influenced by fusion topology, we tested the VN-AbdA/VC-Exd and VN-AbdA/Exd-VC combinations, whose ability to form complexes *in vitro *was reduced. Under the same parameters of image acquisition, BiFC signals resulting from the VN-AbdA/VC-Exd complex assembly were five times weaker than BiFC signals resulting from the VC-AbdA/VN-Exd combination (Figure 5a), while with the VN-AbdA/Exd-VC complex fluorescent signals were visualized only when the sensitivity of the microscope was increased to its maximum (Figure 5b). These results show that the formation of the AbdA/Exd complex was similarly affected by fusion topologies *in vitro *and *in vivo*.

As several protein motifs lying on both sides of the HD of AbdA could potentially interact with Exd [39], it was difficult to predict the best appropriate fusion insertion site in AbdA for BiFC with Exd. In order to test whether the choice of insertion sites could be predicted from the knowledge of the emplacement of known protein interaction domains, we performed BiFC between Exd and its well known nuclear translocation partner Hth. The domains required for Exd-Hth interactions have been well characterized and are located N-terminally in both proteins ([40] and Additional File 3). Thus, we tested the influence of fusion insertions at the N-terminus or C-terminus of Hth (Additional File 3), expecting that the fusion closest to the Exd-interaction domain would more strongly affect BiFC. Surprisingly, we observed the reverse, as it was the C-terminus fusion that produced no BiFC, even under high levels of protein expression and confocal parameters rose at their maximum of sensitivity (Additional File 3).

Altogether, these results highlight the fact that the critical influence of fusion topology on BiFC efficiency is not predictable, even when interacting domains are known, and that the absence of BiFC signals for a given protein complex is difficult to interpret unless different fusion topologies have been tested. However, as shown for AbdA and Exd, previous *in vitro *protein interaction assays like EMSAs can be used for selecting the appropriate fusion topology for further BiFC assays.

### Specificity of BiFC in the living *Drosophila *embryo

In order to be exploitable, all complementation assays are based (with enzymes or fluorescent proteins) on the property of the spontaneous association of split fragments when brought together upon protein interactions [13]. This property explains that the complex formation between fusion proteins is generally stabilized by the association of split fragments, allowing the visualization of weak and transient interactions. Importantly, this property also highlights the need to express fusion proteins at physiological levels and the necessity of using negative controls of BiFC for each protein complex.

In order to analyse the properties of split Venus fragments in the *Drosophila *embryo, we first expressed the VN and VC moieties as such. As expected, when expressed together, these two fragments led to strong BiFC signals which were visible after short incubation times at 4°C (Additional File 4). The level of fluorescence did not increase with longer incubation times (green-dotted curve in Figure 4b), suggesting that the VN/VC association was not regulated, leading to the rapid self-assembly of all VN and VC fragments. The level of fluorescence was stronger at later developmental stages (Additional File 4), providing a background level of unspecific BiFC, and which was probably due to the accumulation of peptide synthesis during embryogenesis.

Self-assembly of Venus moieties did not take place when one of the Venus fragments was fused to a protein, as previously described [21]. Indeed, even after 28 h of incubation, VN/VC-AbdA or VC/VN-AbdA did not produce any BiFC signal or at extremely low levels in late stage embryos (Additional File 4). These results highlight that the affinity between Venus fragments is not strong enough to induce artificial self-assembly in the context of a protein fusion.

Nevertheless, the inherent property of Venus fragments for self-assembly stresses the necessity of performing controls to validate BiFC results. We validated the specificity of BiFC between AbdA and Exd using competition experiments as well as by using mutant fusion proteins impaired in their ability to interact.

**Figure 6:**
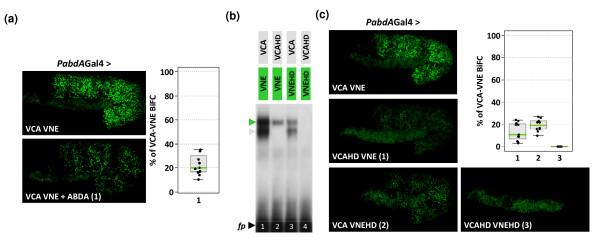
**Specificity of bimolecular fluorescence complementation (BiFC) in the *Drosophila *embryo **. (a) BiFC in live embryos expressing VCA and VNE either alone or with the parental wild type abdominalA (AbdA) protein (combination 1), as indicated. (b-c) Abolishing DNA binding of AbdA and extradenticle (Exd) abolishes heterodimeric complex formation. (b) Electrophoretic mobility shift assays (EMSAs) with wild type or homeodomain (HD)-mutated AbdA and Exd fusion proteins were performed on the *Dll *consensus probe [32]. The HD mutation in one partner strongly affects the heterodimeric complex formation (green arrowhead), while no complex is formed when both partners are mutated. The grey arrowhead indicates AbdA monomer binding. (c) BiFC analysis in embryos expressing various combinations (numbered 1 to 3) of wild-type or HD-mutated fusion proteins, as indicated. All fusion proteins were expressed with the *abdA-Gal4 *driver, and quantifications were performed in stage 10 embryos. See also Additional File 5. Diagrams in (a) and (c) are boxplot representations of the statistical quantification of the surface and intensity of fluorescent signals measured for each indicated combination (numbers in abscises) in the whole embryo.

As VC-AbdA associated less efficiently with VN-Exd than the unfused AbdA protein *in vitro*, we hypothesized that AbdA could act as a competitor against BiFC *in vivo*. This was tested by co-expressing VC-AbdA, VN-Exd and AbdA proteins. We observed that the co-expression led to a loss of 80% of BiFC between VC-AbdA and VN-Exd (Figure 6a), demonstrating that AbdA can outcompete complex formation between VC-AbdA and VN-Exd. This result also shows that complementation between split Venus fragments does not favour the protein complex assembly *in vivo*.

As a second validation control, we searched for a condition where AbdA-Exd interactions could be drastically affected or abolished. As EMSAs previously revealed that AbdA/Exd complex assembly critically depends on monomer DNA binding, we mutated the amino acid 51 of the HD in the VC-AbdA and VN-Exd fusion proteins. These mutations are expected to abolish DNA binding. Accordingly, EMSAs showed that complex formation is strongly affected when one of the two partners contains the HD mutation and that no complex can be formed when both fusion proteins contain the HD mutation (Figure 6b). This result establishes that complex formation between AbdA and Exd cannot occur in the absence of DNA binding *in vitro*, which is not necessarily the case for interactions between AbdA and other cofactors (see below). The effect of HD mutations was next analysed on BiFC in the *Drosophila *embryo. We verified that HD-mutated fusion proteins were properly expressed, at levels equivalent to the wild type fusion proteins (Additional File 5). BiFC with the *abdA-Gal4 *driver showed that combinations involving one partner protein mutated in the HD produced weak fluorescent signals (Figure 6c). No BiFC was obtained when both fusion proteins were mutated (Figure 6c), even when expressed at higher levels with the *engrailed (en)-Gal4 *driver (Additional File 5). Thus, BiFC with the HD-mutated forms of AbdA and Exd recapitulates the previous *in vitro *observations.

Altogether, these data establish that BiFC cannot occur in the absence of AbdA-Exd interactions and that the complementation between split Venus fragments is not sufficient to promote artificial protein complex assembly.

### BiFC can reveal a spatial control of protein interactions

**Figure 7:**
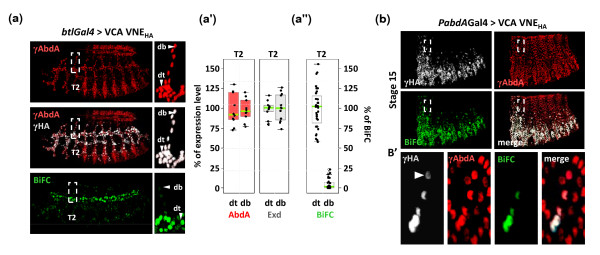
**Bimolecular fluorescence complementation (BiFC) can reveal a spatial control of protein interactions in the *Drosophila *embryo**. (a) Spatial control of the BiFC signal resulting from the VCA/VNE complex assembly in the tracheal system. Despite the uniform expression of abdominalA (AbdA; anti-AbdA, red) and extradenticle (Exd; anti-HA, grey) fusion proteins in all tracheal branches with the *breathless (btl)-Gal4 *driver, BiFC is mostly apparent in the dorsal trunk (dt) and strikingly weaker in other tracheal branches. Enlargements (dotted-white boxes) focused on this discrepancy in the T2 segment between the dt and the dorsal branch (db). Note that endogenous AbdA protein is not present in this segment. (a') Statistical quantification of the VCA (red-filled boxplot) and VNE (grey-filled boxplot) expression levels in the dt and db of the T2 segment, as deduced from immunostainings. (a'') Despite comparable expression levels of VCA and VNE fusion proteins in the two tracheal branches, BiFC is statistically lower in the db than in the dt. A total of five nuclei in ten different embryos were quantified in the two corresponding branches. (b-b') Spatial dynamics of the BiFC signal in the dorsal epidermis of stage 15 embryos. Expression of fusion proteins (as revealed with anti-HA that specifically recognizes the HA-tagged VNE fusion protein, grey) in embryos heterozygous for the *abdA-Gal4 *driver is progressively lost in dorsal most parts of the epidermis, while endogenous AbdA (red, as revealed with an anti-AbdA antibody) continues to be homogeneously expressed. BiFC signal (green) follows the dynamic of the *abda-Gal4 *driver since it is no longer observed in dorsal most parts of the embryo, even when fusion proteins are not completely absent (as revealed with the anti-HA: white arrowhead in b', corresponding to the enlargement of white-dotted boxes in b).

BiFC might serve to reveal spatially controlled protein interactions. Thus, we searched for contexts where the AbdA/Exd complex assembly could be regulated. This was achieved by expressing the VC-AbdA and VN-Exd fusion proteins in different embryonic tissues with the help of specific *Gal4 *drivers. We found that the interaction pattern of VC-AbdA/VN-Exd was spatially controlled within the tracheal system. Indeed, although fusion proteins are homogeneously expressed within all tracheal branches (as shown for two tracheal branches in the T2 segment, where no endogenous AbdA protein is present, Figures 7a-a'), BiFC is mostly visible in a main tracheal branch, the dorsal trunk (dt; quantification is shown in Figure 7a''). BiFC thus reveals the existence of a spatial control promoting AbdA/Exd assembly in the dt and/or avoiding AbdA/Exd interactions in other tracheal branches like the dorsal branch (db). This specific interaction profile also demonstrates that the inherent tendency of split Venus fragments to self associate is not strong enough to promote artificial protein complex assembly in the context of regulated protein-protein interactions.

Since the association between split fragments of Venus is likely to stabilize protein interactions, as noticed *in vitro *[6], we analysed whether this could extend the life time of the AbdA/Exd complex. This issue was addressed by taking advantage of the *abdA-Gal4 *driver which is progressively turned off during late embryonic stages in dorsal-most epidermis, while endogenous AbdA remains unchanged (Figures 7b-b'). We hypothesized that stabilisation of heterodimer complex formation by fluorescence complementation will lead to BiFC signals that will not follow the temporal dynamic of *abdA-Gal4 *driver expression, and will rather mimic the sustained endogenous AbdA expression pattern. This was clearly not the case since we observed loss of BiFC in dorsal most parts of late stage embryos, even for weak expression levels of fusion proteins (as assessed with the anti-HA staining against the VN-Exd-HA-tagged construct: Figures 7b-b'). This result shows that BiFC detection accurately follows dynamics of fusion proteins expression and/or degradation.

### Multicolour BiFC in the *Drosophila *embryo

**Figure 8:**
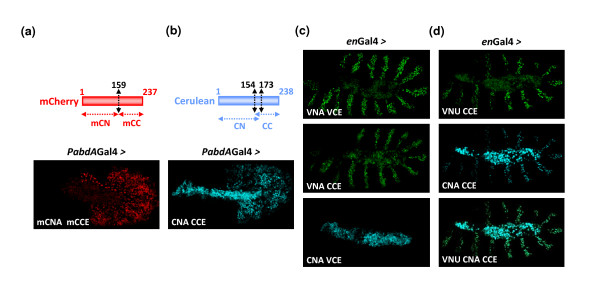
**Multicolour bimolecular fluorescence complementation (BiFC) and simultaneous visualization of multiple protein interactions**. (a) BiFC with the red fluorescent protein mCherry. Position of the cut is indicated and was chosen according to previous works in cell cultures [9]. The abdominalA (AbdA) and extradenticle (Exd) fusion proteins were generated with the N-terminal (mCN) or C-terminal (mCC) fragment of mCherry, respectively. BiFC was visualized with the *abdA-Gal4 *driver. (b) BiFC with the blue fluorescent protein Cerulean. Split Cerulean fragments were generated as in Venus. The AbdA and Exd fusion proteins were constructed with the N-terminal (CN) or C-terminal (CC) fragment of Cerulean, respectively. BiFC was visualized with the *abdA-Gal4 *driver. (c) Complementation between split fragments of the Venus and Cerulean fluorescent proteins. The VN and CC fragments are able to complement, producing a Venus-like fluorescent signal that is weaker than the one obtained between split fragments of Venus. The CN and VC fragments do not produce BiFC signals, as previously described in cell cultures [7]. Fusion proteins were expressed with the *engrailed (en)-Gal4 *driver. (d) Multicolour BiFC between ultrabithorax (Ubx), AbdA and Exd proteins fused to split fragments of the Venus and Cerulean proteins, as indicated. All fusion proteins are expressed simultaneously with the *en-Gal4 *driver. Images of live embryos were acquired separately with specific filters (see Methods). Note that the fluorescence in the middle of the embryo is not specific and corresponds to the auto-fluorescence of the amnioserosa. Auto-fluorescence is particularly strong in the Cerulean spectrum.

In order to assess whether multicolour BiFC could be applied in the *Drosophila *embryo, the suitability of the red mCherry [9] and blue Cerulean [11] fluorescent proteins was assayed. Split mCherry and Cerulean fragments were fused to the N-terminus of AbdA and Exd (Figures 8a and 8b), reproducing fusion topologies found to be exploitable with split Venus fragments. Again, the expression level of fusion constructs was verified to be close to endogenous AbdA expression levels (not shown). The red and blue fluorescent signals were visualised following their respective excitation wave lengths (see Methods). We found that BiFC with mCherry and Cerulean was weaker than BiFC with Venus (Figures 8a and 8b). This was mainly due to a weaker brightness of mCherry and Cerulean but it was also due to a higher level of non-specific fluorescent background in the Cerulean emission spectra (particularly in the amnioserosa).

Nevertheless, BiFC with Cerulean appears to be exploitable in the *Drosophila *embryo. This offers the possibility to test for multicolour BiFC, allowing the simultaneous visualization of two different protein complexes. This property is based on the capacity of the VN fragment to complement with the C-terminal fragment of Cerulean, producing a Venus-like fluorescent signal (Figure 8c and [7]). Multicolour BiFC was tested by co-expressing two Hox proteins, Ultrabithorax (Ubx) and AbdA, fused respectively to the N-terminal fragment of Venus (VN) or Cerulean (CN), with the Exd protein fused to the C-terminal fragment of Cerulean (CC). Expression of these three fusion proteins led to simultaneous Venus and Cerulean fluorescent signals in the same nuclei, corresponding to the VN-Ubx/CC-Exd and CN-AbdA/CC-Exd complexes, respectively (Figure 8d). Of note, these fusion proteins were expressed at twice their physiological level with an *engrailed-Gal4 *driver to thwart the possible competition of endogenous proteins. This result establishes that multicolour BiFC can be performed in the *Drosophila *embryo for simultaneous visualization of multiple protein interactions.

### Suitability of BiFC for *in vivo *identification of candidate interacting partners

The role of the PBC class proteins as Hox cofactors is well established. However, several Hox functions are PBC-independent, and other factors have been reported to interact with Hox proteins [41] and/or to assist them in the regulation of common downstream target genes [24]. One class of such protein partners that has long been postulated comprises Hox proteins themselves. For example, Hox-Hox interactions have been proposed to occur in *Drosophila *to explain a phenomenon of quantitative competition and mutually inhibitory functions [42]. Interactions among different vertebrate Hox proteins have also been described *in vitro *[43].

Here, we took advantage of BiFC to directly address the interaction status between AbdA and different candidate interacting proteins *in vivo*. Of note, all candidate interacting proteins were fused to the VN fragment, allowing analysis with VC-AbdA, which we previously showed as best suited for BiFC analysis. The position of the VN fragment in fusion proteins was dictated by cloning constraints since the best appropriate fusion topology cannot be predicted, as demonstrated above.

**Figure 9:**
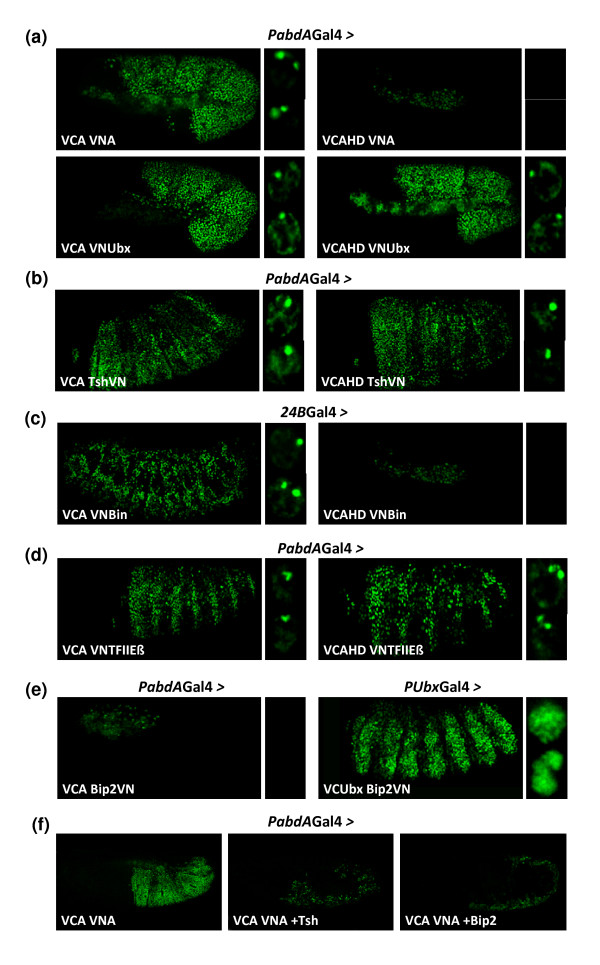
**Suitability of bimolecular fluorescence complementation (BiFC) for *in vivo *identification of interacting partners **(a) BiFC resulting from abdominalA (AbdA)-AbdA and AbdA-ultrabithorax (Ubx) interactions. The homeodomain (HD) mutation in AbdA abolishes homodimeric complex formation only. (b) BiFC between AbdA and the transcription factor Teashirt (Tsh). The HD mutation in AbdA does not abolish AbdA/Tsh complex formation. (c) BiFC between AbdA and the transcription factor Biniou (Bin). Fusions proteins were expressed in the mesoderm. The HD mutation in AbdA abolishes AbdA/Bin complex formation. (d) BiFC between AbdA and the basal transcription machinery protein TFIIbeta. The HD mutation in AbdA does not abolish AbdA/TFIIbeta complex formation. (e) BiFC between AbdA or Ubx and the basal transcription machinery protein BIP2. No BiFC can be visualized between AbdA and BIP2. (f) Competition experiments against BiFC resulting from the VCA/VNA complex assembly. Simultaneous expression of Tsh or BIP2 drastically affects BiFC, suggesting that these two partners interact with AbdA fusion proteins, thereby titrating BiFC complexes. Candidate interacting partners are fused to the VN fragment as indicated, and drivers used are written above pictures. Enlargements on the right focused on the BiFC profile within nuclei.

We first verified the *in vivo *existence of Hox-Hox interactions for AbdA and observed that it is able to form homodimeric complexes (Figure 9a). In addition, AbdA can also form heterodimeric complexes with Ubx (Figure 9a). Interestingly, the HD mutation of AbdA affected homodimeric but not heterodimeric complex formation (Figure 9a). Although these interactions behave differently with regard to DNA-binding dependency, they both present a similar nuclear localization, with BiFC occurring systematically at two nuclear loci (Figure 9a), suggesting a tight sub-nuclear control of Hox-Hox interactions.

We next investigated whether proteins previously described as putative Hox cofactors could indeed directly interact with AbdA. Candidate proteins were chosen on the basis of genetic and/or biochemical analysis but direct evidence for *in vivo *interactions with AbdA had not been established. They were also chosen to cover distinct functions, such as region- or tissue-specific transcription factors or proteins of the basal transcription machinery.

We first tested Teashirt (Tsh), which is a zinc finger transcription factor. Tsh has been genetically described to act with Antennapedia (Antp) and Hox proteins of the Bithorax complex for specifying the embryonic trunk region [44]. More recently, Tsh was also shown to directly interact with Sex combs reduced for specifying the prothorax in *Drosophila *[45]. We observed that the VC-AbdA/Tsh-VN complex produced strong BiFC signals (Figure 9b), indicating that Tsh acts through direct interactions with AbdA. Moreover, AbdA/Tsh complexes accumulate in a few nuclear foci, suggesting that their assembly is tightly regulated within the nucleus (Figure 9b). Finally, BiFC with the HD-mutated form of AbdA showed that AbdA-Tsh interactions do not depend on AbdA DNA-binding (Figure 9b).

The second putative partner analysed is a tissue-specific transcription factor, Biniou (Bin), which contains a forkhead DNA-binding domain. Bin has been proposed to act as a Hox cofactor for the regulation of several target genes in the visceral mesoderm [46]. BiFC, indeed, showed that AbdA interacts with Bin in few nuclear loci (Figure 9c) and in a DNA-binding-dependent manner (Figure 9c).

Finally, we tested the interaction potential of AbdA with two candidate proteins belonging to the basal transcriptional machinery, TFIIbeta and BIP2. TFIIbeta has been shown to interact with Ubx in a yeast two-hybrid assay [41], while BIP2 has been shown to interact with Antp [47]. We found that AbdA/TFIIbeta (Figure 9d), but not AbdA/BIP2 complexes (Figure 9e), produced BiFC signals. Fluorescent signals were not affected upon the HD mutation in AbdA, suggesting that AbdA-TFIIbeta interaction did not depend on AbdA DNA-binding (Figure 9d).

Our results raised two limits of the BiFC for a candidate interaction screening. First, even if we observed that BIP2-VN was able to produce strong BiFC signals with VC-Ubx (Figure 9e), the absence of BiFC between VC-AbdA and BIP2-VN is not conclusive, since it could simply result from unfavourable fusion topologies, as previously described. Secondly, we noticed that the HD mutation in AbdA, which abolished interactions with Exd, did not affect BiFC with some candidate partners. This stresses the difficulty of finding appropriate negative controls when molecular cues required for protein complex assembly are not known. As already mentioned, competition experiments can provide suitable controls. We reasoned that specific interactions should be competitive against BiFC resulting from the VC-AbdA/VN-AbdA complex assembly, which can thus be used as an experimental test to validate interactions between AbdA and a given protein partner.

In order to validate the observed BiFC between AbdA and Tsh, we co-expressed VC-AbdA and VN-AbdA with wild-type Tsh, which we designed as a 'cold' competitive partner (not fused to a Venus fragment). We observed that this co-expression led to a drastic loss of BiFC (Figure 9f), which can be explained by the titration of VC-AbdA/VN-AbdA dimers outcompeted by cold (non fluorescent) VC-AbdA/Tsh and VN-AbdA/Tsh complexes.

The same type of experiment was performed with BIP2 to validate absence of BiFC between AbdA and BIP2. We observed that co-expressing the cold wild-type BIP2 protein led to a strong decrease of BiFC resulting from the VC-AbdA/VN-AbdA complex assembly (Figure 9f). We concluded that BIP2 can interact with AbdA despite unproductive BiFC assay, which in turn suggests that, in contrast to Tsh, BiFC with BIP2 was not visualized because of unfavourable fusion topologies.

## Discussion

### Establishing BiFC as a specific protein interaction assay in the *Drosophila *embryo

By using Hox partnership as a paradigm, we have demonstrated the suitability of BiFC for the study of transcription factors interactions in the *Drosophila *embryo. Our extensive analysis highlights the need for careful selection of fusion topologies, which is of particular importance when considering that absence of BiFC signal can simply result of an unfavourable fusion topology. There is no simple rule for predicting the arrangement of fusion topologies that will produce maximal signals, even when the protein interaction domains are known. For example, in the case of Hth, the N-terminus Exd interaction domain was paradoxically more affected by fusions at the C-terminus of the protein. Similarly, the best fusion topologies in AbdA and Exd cannot be predicted, although Hox/PBC interfaces have been well characterized by structural studies. Since these structures were obtained with portions of proteins, it can simply not be informative in the context of the three dimensional arrangements of the full length protein. Appropriate fusion constructs can, however, be rapidly identified with the help of *in vitro *interaction assays, limiting the number of *Drosophila *transgenic lines that need to be generated. In the case of AbdA and Exd, we showed that EMSA provides a mean to select the best suited fusion proteins for subsequent BiFC analysis in the *Drosophila *embryo. The impact of fusion topologies on BiFC also underlines the need of using the same fusion topology when comparing protein interaction efficiencies.

We showed that the VN and VC fragments were able to self assemble with a typical kinetic when expressed as single peptides. Although this property was expected for a visualisation method based on complementation, it also emphasized the need of control experiments for validating BiFC results. Here, we provided several evidences demonstrating the specificity of BiFC for the study of protein interactions in the *Drosophila *embryo. This includes the extremely weak tendency of VN and VC fragments to self-associate when one fragment is fused to AbdA, the destabilization of complex formation through protein competition, the absence of BiFC signals in absence of interactions, the spatial restriction of BiFC in the tracheal system or absence of BiFC between AbdA and BIP2.

We found that the time required for visualizing BiFC signals can be provided by keeping embryos at 4°C, ideally for a period no longer than 28 h. This procedure allows the visualization of protein interactions within the short time window of *Drosophila *embryonic development and suggests that the methodology could be generally suited for the study of protein interactions in the context of other rapid developmental processes. Finally, we showed that multicolour BiFC is applicable to the *Drosophila *embryo, extending further the potential of the methodology for *in vivo *simultaneous study of multiple protein interactions.

### BiFC, a simple and sensitive method allowing *in vivo *analyses of protein interactions under physiological conditions

BiFC is a commonly used method in cultured cells and in plants for studying protein interactions. Surprisingly, this method remains rarely used in living animal embryos. In contrast, the visualization method based on fluorescence resonance energy transfer (FRET) has been more thoroughly used in animal models, although it requires complex instrumentation and data processing. FRET analysis is based on energy transfer between two fluorophores placed in close proximity, allowing real time detection of complex formation and dissociation. The detection of this energy transfer usually requires high levels of protein expression [13] and is crucially dependent of the distance between the donor and acceptor molecules [48]. This constrain may explain that FRET is mainly used to monitor conformational changes, processes of great importance in signalling [49]. Accordingly, FRET has only been used in the context of biosensors [50,51] and as a caspase activity indicator [52] in *Drosophila*.

In this work, we establish BiFC as a complementary approach to FRET. We show that the sensitivity of BiFC enables it to work at physiological levels of expression. This is a crucial property of the methodology, since quantitative differences in protein interactions play important roles in controlling *in vivo *protein functions. In order to validate BiFC as a unique visualization method for measuring physiologically relevant protein interactions, we have generated a *P-Gal4 *driver by replacing a *P-lacZ *insertion in *abdA *cis-regulatory sequences. This driver reproduces the *abdA *expression profile and we selected experimental conditions allowing expression levels close to endogenous AbdA. Importantly, this insertion affects endogenous *abdA *expression, which offers the advantage of performing BiFC in cells containing normal doses of AbdA. Loss of the endogenous competitive protein can also be crucial for visualising weak protein interactions by BiFC. The huge number of genomic *P *insertions in *Drosophila *are thus of great utility for performing BiFC under physiological conditions.

### Identification of interacting protein partners using BiFC

Given the sensitivity, specificity and simplicity of BiFC, the method should in principle be suited for the identification of interacting protein partners. We investigated the suitability of the method for revealing protein interactions of different natures, using a candidate gene approach. Although the expression level of all candidate cofactors was not systematically verified, several transgenic lines were tested in each case. This includes: Hox-Hox interactions that do not involve a TALE (three amino acids loop extension) homeodomain as in the case of AbdA/Exd interactions; interactions with proteins of the basal transcription machinery; and interactions with zinc-finger or forkhead transcription factors. The nature of these interacting partners, involved in different transcriptional processes, illustrates the potential of Hox proteins to accommodate interactions with various classes of transcription factors.

We tried to validate the specificity of these interactions by using a DNA-binding deficient form of AbdA. We observed that this mutation abolished interactions with Exd or Bin but had no effect on interactions with Ubx, Tsh or TFIIbeta. This result stresses that the DNA binding of Hox proteins is not a mandatory step for recruiting protein partners *in vivo*. It also illustrates the difficulty of validating BiFC observations by using mutated proteins when the molecular cues required for the complex assembly are not known. We showed that an alternative way to validate BiFC observations is through competition experiments. This was achieved by co-expressing the candidate partner as a 'cold' protein with the VC-AbdA and VN-AbdA fusion proteins. In the context of specific interactions, the titration of AbdA fusion proteins by the cold competitive partner will affect BiFC resulting from the VC-AbdA/VN-AbdA complex assembly, as observed with Tsh. The same approach can also be instructive in absence of BiFC, as shown with BIP2, revealing an unfavourable arrangement of fusion topologies for visualising protein interactions.

Interestingly, interactions between AbdA and candidate partners have distinct and typical nuclear localisations, suggesting that the nuclear architecture has important roles in controlling Hox protein interactions and functions. It also illustrates the power of BiFC for deciphering unanticipated mechanisms regulating protein interactions within a proper physiological context.

Finally, the variety of protein interactions visualized through BiFC indicates that the methodology could be used more globally [53] for screening for novel interacting partners in a developing organism. This could readily be achieved combining BiFC with the strength of *Drosophila *genetics.

## Conclusions

We have explained how to perform multicolour BiFC with physiological levels of protein expression in the live *Drosophila *embryo. We provided experimental procedures to circumvent the limitations of BiFC and we designed control experiments to validate BiFC results. We also revealed the importance of choosing the best match of fusion proteins, which can be rapidly achieved with transcription factors through EMSA experiments. Finally, we described how to use BiFC for finding novel cofactors through a candidate gene approach. Altogether, our work demonstrates that BiFC is not only a physiologically relevant protein interaction test but also a powerful method for studying protein interaction dynamics during *Drosophila *embryogenesis.

## Abbreviations

AbdA: abdominalA; BiFC: bimolecular fluorescence complementation; CC: C-terminal fragment of Cerulean; CFP: cyan fluorescent protein; CN: N-terminal fragment of Cerulean; dt: dorsal trunk; EMSA: electrophoretic mobility shift assay; Exd: extradenticle; FRET: fluorescence resonance energy transfer; GFP: green FP; HD: homeodomain; Hth: homothorax; TALE: three amino acids insertion; PCR: polymerase chain reaction; Tsh: Teashirt; Bin: biniou; Ubx: ultrabithorax; YFP: yellow FP; VN: Venus; VN: N-terminal fragment of venus; VC: C-terminal fragment of Venus; mCN: N-terminal fragment of mCherry; mCC: C-terminal fragment of mCherry

## Authors' contributions

BH performed most of the experiments. SM conceived the project, designed most of the experiments and performed some of them. SV helped in the performance of some of the experiments. BH, YG and SM wrote the paper together.

## Supplementary Material

Additional File 1 **Establishing physiological levels of protein expression with the *ultrabithorax (Ubx)-Gal4 *driver**. (A) The *Ubx-Gal4 *driver was used to express the green fluorescent protein (GFP) reporter protein (red), showing an expression profile similar to endogenous Ubx protein (grey) in a stage 10 embryo. (B) Establishing physiological levels of VC-Ubx (VCU) expression with the *armadillo (arm)-Gal4 *driver. The average level of VCU was quantified in the T2 thoracic segment and compared to the level of endogenous Ubx in the A1 segment of a wild type embryo (red-dotted circles). Fluorescent immunostainings were similarly performed with an anti-Ubx antibody (grey). Graph on the right is a boxplot representation of the statistical quantification of the surface and intensity of the fluorescent Ubx immunostaining. It shows that VCU is expressed at around 80% of endogenous Ubx under these conditions. (C) Establishing physiological levels of expression with the *Ubx-Gal4 *driver. Quantifications were measured with an anti-GFP that recognizes the VC fragment of VCU. Fluorescent immunostainings (grey) were performed in embryos expressing VCU either with *arm-Gal4 *or *Ubx-Gal4 *at 29°C. Graph on the right indicates that *Ubx-Gal4 *led to a slightly better expression than *armGal4 *(around 20% more). From (B) and (C), we concluded that using *Ubx-Gal4 *at 29°C allows expression levels comparable to endogenous Ubx levels found in the A1 segment of a wild type embryo. Click here for file

Additional File 2 **Additional File 2**. Live imaging of a developing embryo expressing the VC-abdominalA (AbdA) and VN-extradenticle (Exd) fusion proteins with the *abdA-Gal4 *driver. Live imaging was acquired from stage 10 to stage 14 of embryogenesis. Click here for file

Additional File 3 **Influence of fusion topologies on bimolecular fluorescence complementation (BiFC) resulting from extradenticle (Exd)/homothorox (Hth) complex assembly**. (A) Schematic representation of Exd and Hth fusion proteins. Interacting domains (PBCA in Exd, HM in Hth) are indicated. (B) BiFC with the indicated fusion proteins which were expressed with the *engrailed (en)-Gal4 *driver. No signal can be visualized between Hth-VN and VC-Exd. (C) The VC-Hth and Hth-VN fusion proteins are expressed at similar levels with the *en-Gal4 *driver. Fusion proteins expression was revealed with a polyclonal anti-green fluorescent protein antibody (grey) that recognizes both fragments of Venus. Images were acquired with identical confocal parameters. Click here for file

Additional File 4 **Self-assembly properties of the VN and VC fragments in the *Drosophila *embryo**. (A) The VN and VC fragments were expressed with the *abdA-Gal4 *driver, either as isolated peptides, or in the context of an abdominalA fusion protein, as indicated above pictures. Bimolecular fluorescence complementation (BiFC) was visualized in stage 11 or stage 14 embryos, after 28 h of incubation at 4°C. BiFC resulting from the assembly of isolated Venus (VN) and VC fragments was already visible after a short incubation time of 2 h, but the intensity of the fluorescence did not increase with longer times of incubation (see also the green-dotted curve in Figure 4b). (B) Expression level of the VN and VC fragments, as revealed with a polyclonal anti-green fluorescent protein antibody (grey) that recognizes both fragments. Images were acquired with identical confocal parameters. Note that the VN fragment is more specifically addressed to the nucleus than the VC fragment, due to the addition of a nuclear localization signal (see Methods). Click here for file

Additional File 5 **The mutation in the homeodomain (HD) of abdominalA (AbdA) and extradenticle (Exd) does not affect their expression profile and abolishes bimolecular fluorescence complementation (BiFC)**. (A) The wild-type (VCA) and homeodomain (HD)-mutated (VCAHD) forms of the VC-AbdA fusion protein are expressed at comparable levels in the embryo. Quantifications were performed with an anti-green fluorescent protein antibody (grey) in embryos heterozygous for the *PabdAGal4 *driver. (B) The wild-type (VNE) and HD-mutated forms (VNEHD) of the HA-tagged VN-Exd fusion proteins are expressed at comparable levels in the embryo. Quantifications were performed with an anti-HA antibody (grey) in embryos heterozygous for the *PabdAGal4 *driver. Graphs on the right illustrate the statistical quantification as boxplot. (C) The VCAHD and VNEHD fusion proteins did not produced BiFC *in vivo*. Fusion proteins were expressed with the *engrailed (en)-Gal4 *driver at 18°C or 29°C. High levels of fusion proteins expression were confirmed by the AbdA (magenta) and Exd (with anti-HA, grey) immunostainings. Despite these high levels of protein expression, no BiFC can be visualized (upper images), highlighting the specificity of the methodology. Click here for file
