# Supplementary material for: Visualization of protein interactions in living Drosophila embryos by the bimolecular fluorescence complementation assay
Source: BMC Biol. 2011 Jan 28;9:5. doi: 10.1186/1741-7007-9-5 (PMC3041725; doi:10.1186/1741-7007-9-5)
Supplement: Additional File 1 — Establishing physiological levels of protein expression with the ultrabithorax (Ubx)-Gal4 driver. (A) The Ubx-Gal4 driver was used to express the green fluorescent protein (GFP) reporter protein (red), showing an expression profile similar to endogenous Ubx protein (grey) in a stage 10 embryo. (B) Establishing physiological levels of VC-Ubx (VCU) expression with the armadillo (arm)-Gal4 driver. The average level of VCU was quantified in the T2 thoracic segment and compared to the level of endogenous Ubx in the A1 segment of a wild type embryo (red-dotted circles). Fluorescent immunostainings were similarly performed with an anti-Ubx antibody (grey). Graph on the right is a boxplot representation of the statistical quantification of the surface and intensity of the fluorescent Ubx immunostaining. It shows that VCU is expressed at around 80% of endogenous Ubx under these conditions. (C) Establishing physiological levels of expression with the Ubx-Gal4 driver. Quantifications were measured with an anti-GFP that recognizes the VC fragment of VCU. Fluorescent immunostainings (grey) were performed in embryos expressing VCU either with arm-Gal4 or Ubx-Gal4 at 29°C. Graph on the right indicates that Ubx-Gal4 led to a slightly better expression than armGal4 (around 20% more). From (B) and (C), we concluded that using Ubx-Gal4 at 29°C allows expression levels comparable to endogenous Ubx levels found in the A1 segment of a wild type embryo. [file 1741-7007-9-5-S1.ppt]

## Slide 1
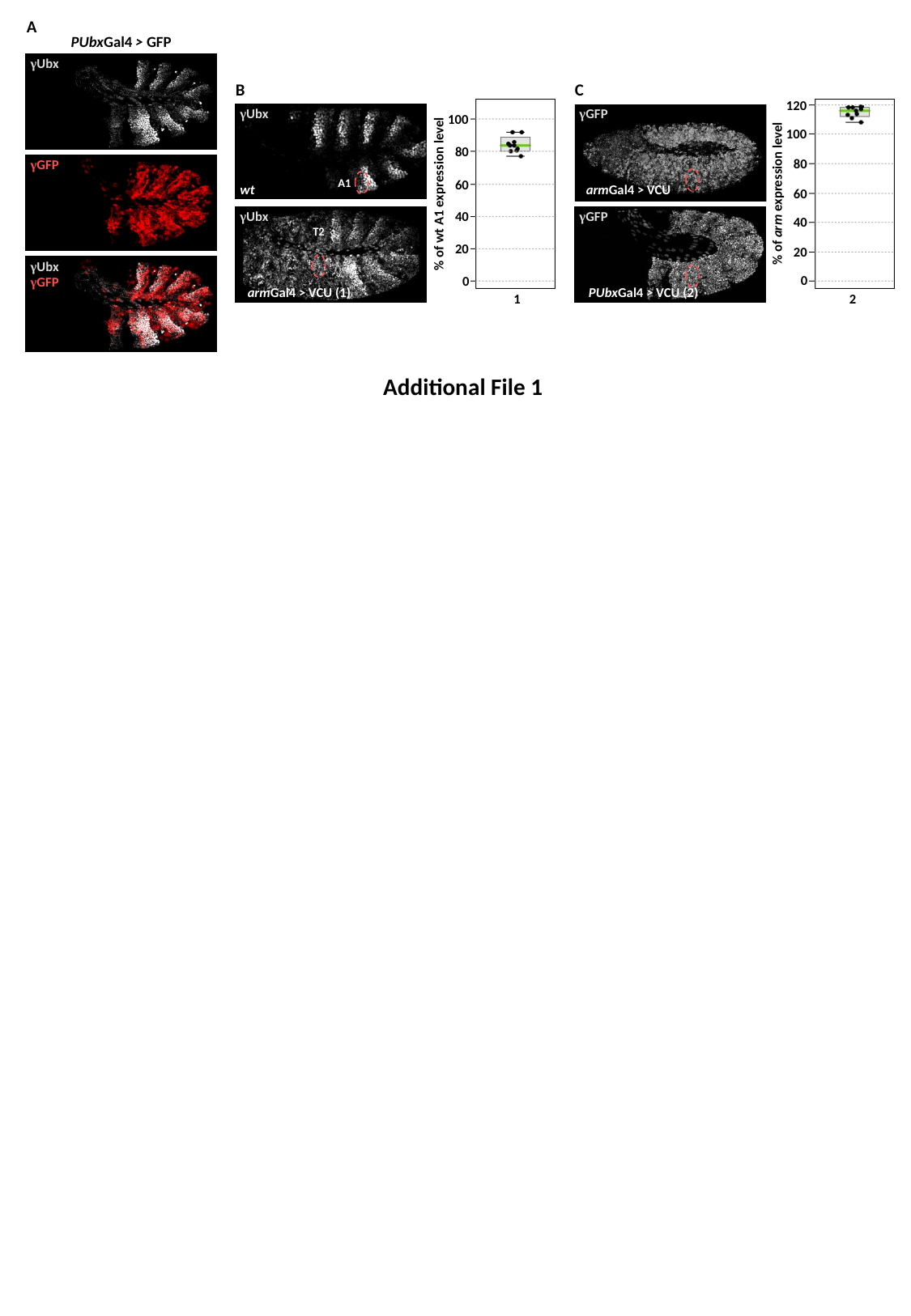

A
PUbxGal4 > GFP
γUbx
B
C
120
γUbx
γGFP
100
100
80
80
γGFP
A1
60
wt
armGal4 > VCU
60
% of arm expression level
% of wt A1 expression level
γUbx
40
γGFP
40
T2
20
20
γUbx γGFP
0
0
armGal4 > VCU (1)
PUbxGal4 > VCU (2)
2
1
Additional File 1
