# Supplementary material for: Visualization of protein interactions in living Drosophila embryos by the bimolecular fluorescence complementation assay
Source: BMC Biol. 2011 Jan 28;9:5. doi: 10.1186/1741-7007-9-5 (PMC3041725; doi:10.1186/1741-7007-9-5)
Supplement: Additional File 3 — Influence of fusion topologies on bimolecular fluorescence complementation (BiFC) resulting from extradenticle (Exd)/homothorox (Hth) complex assembly. (A) Schematic representation of Exd and Hth fusion proteins. Interacting domains (PBCA in Exd, HM in Hth) are indicated. (B) BiFC with the indicated fusion proteins which were expressed with the engrailed (en)-Gal4 driver. No signal can be visualized between Hth-VN and VC-Exd. (C) The VC-Hth and Hth-VN fusion proteins are expressed at similar levels with the en-Gal4 driver. Fusion proteins expression was revealed with a polyclonal anti-green fluorescent protein antibody (grey) that recognizes both fragments of Venus. Images were acquired with identical confocal parameters. [file 1741-7007-9-5-S3.pptx]

## Slide 1
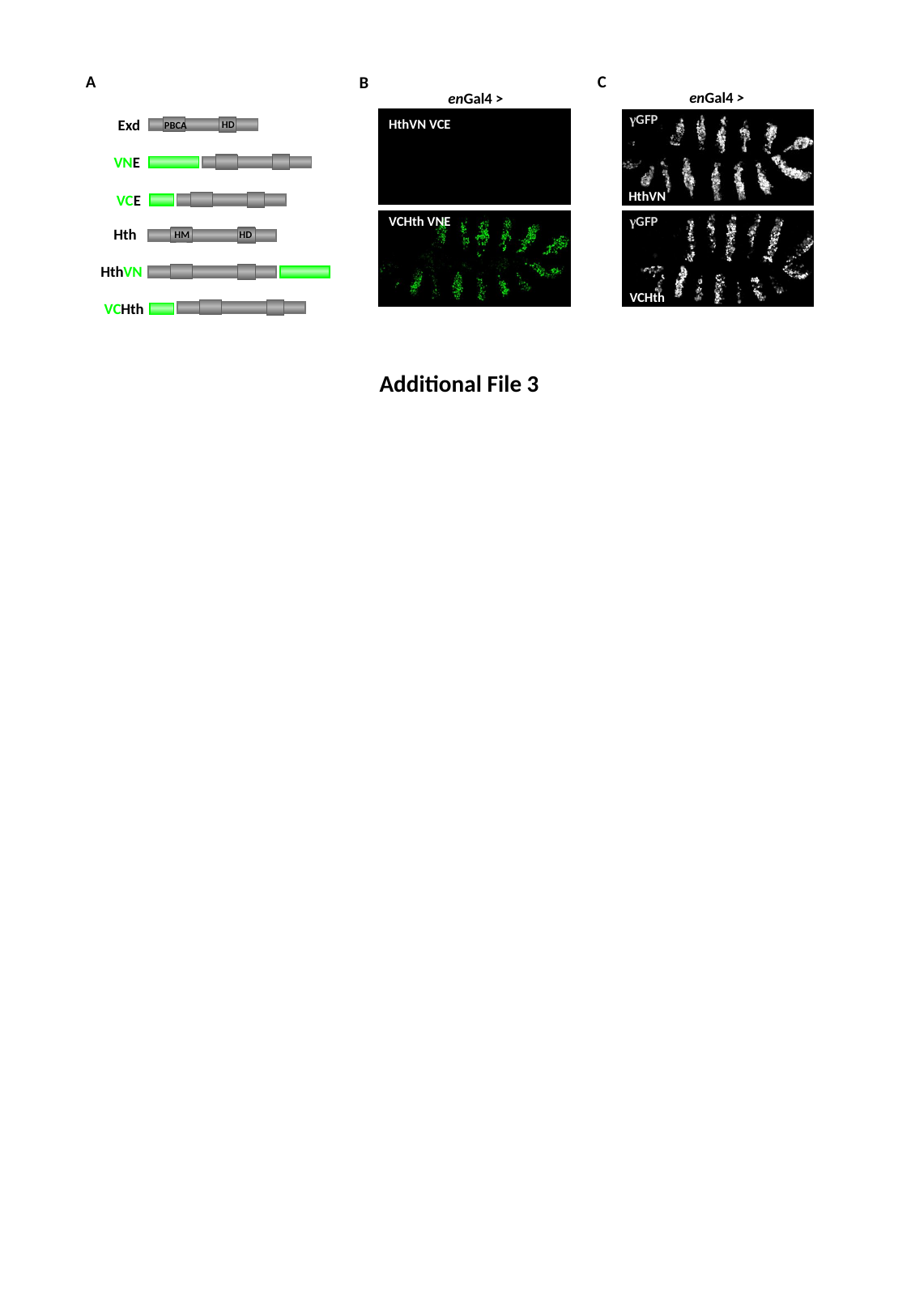

A
C
B
enGal4 >
enGal4 >
γGFP
Exd
HD
PBCA
VNE
VCE
HthVN VCE
HthVN
VCHth VNE
γGFP
Hth
HM
HD
HthVN
VCHth
VCHth
Additional File 3
