# Supplementary material for: Visualization of protein interactions in living Drosophila embryos by the bimolecular fluorescence complementation assay
Source: BMC Biol. 2011 Jan 28;9:5. doi: 10.1186/1741-7007-9-5 (PMC3041725; doi:10.1186/1741-7007-9-5)
Supplement: Additional File 4 — Self-assembly properties of the VN and VC fragments in the Drosophila embryo. (A) The VN and VC fragments were expressed with the abdA-Gal4 driver, either as isolated peptides, or in the context of an abdominalA fusion protein, as indicated above pictures. Bimolecular fluorescence complementation (BiFC) was visualized in stage 11 or stage 14 embryos, after 28 h of incubation at 4°C. BiFC resulting from the assembly of isolated Venus (VN) and VC fragments was already visible after a short incubation time of 2 h, but the intensity of the fluorescence did not increase with longer times of incubation (see also the green-dotted curve in Figure 4b). (B) Expression level of the VN and VC fragments, as revealed with a polyclonal anti-green fluorescent protein antibody (grey) that recognizes both fragments. Images were acquired with identical confocal parameters. Note that the VN fragment is more specifically addressed to the nucleus than the VC fragment, due to the addition of a nuclear localization signal (see Methods). [file 1741-7007-9-5-S4.pptx]

## Slide 1
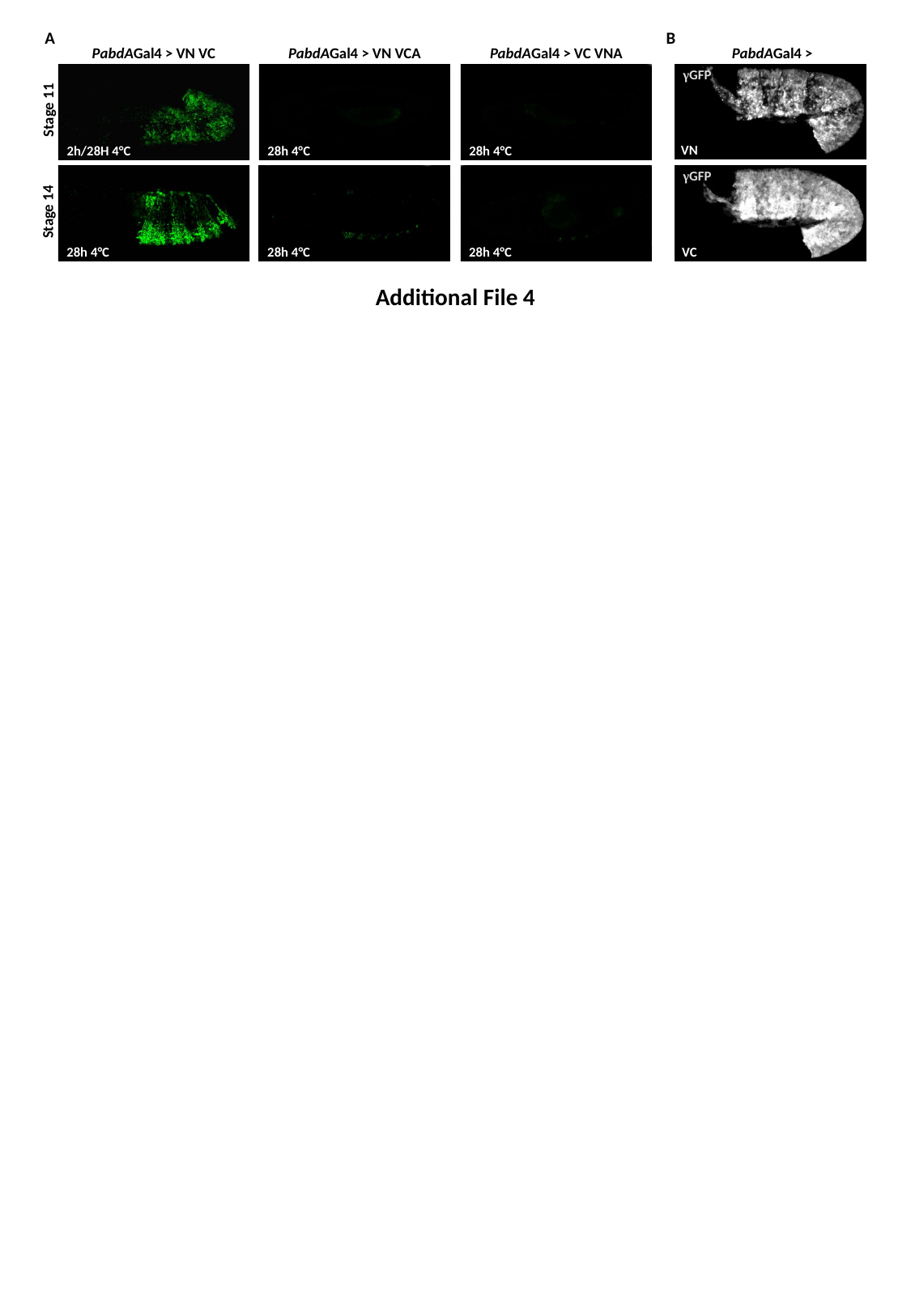

A
B
PabdAGal4 > VN VC
PabdAGal4 > VN VCA
PabdAGal4 > VC VNA
PabdAGal4 >
γGFP
Stage 11
VN
2h/28H 4°C
28h 4°C
28h 4°C
γGFP
Stage 14
28h 4°C
28h 4°C
28h 4°C
VC
Additional File 4
