# Supplementary material for: Visualization of protein interactions in living Drosophila embryos by the bimolecular fluorescence complementation assay
Source: BMC Biol. 2011 Jan 28;9:5. doi: 10.1186/1741-7007-9-5 (PMC3041725; doi:10.1186/1741-7007-9-5)
Supplement: Additional File 5 — The mutation in the homeodomain (HD) of abdominalA (AbdA) and extradenticle (Exd) does not affect their expression profile and abolishes bimolecular fluorescence complementation (BiFC). (A) The wild-type (VCA) and homeodomain (HD)-mutated (VCAHD) forms of the VC-AbdA fusion protein are expressed at comparable levels in the embryo. Quantifications were performed with an anti-green fluorescent protein antibody (grey) in embryos heterozygous for the PabdAGal4 driver. (B) The wild-type (VNE) and HD-mutated forms (VNEHD) of the HA-tagged VN-Exd fusion proteins are expressed at comparable levels in the embryo. Quantifications were performed with an anti-HA antibody (grey) in embryos heterozygous for the PabdAGal4 driver. Graphs on the right illustrate the statistical quantification as boxplot. (C) The VCAHD and VNEHD fusion proteins did not produced BiFC in vivo. Fusion proteins were expressed with the engrailed (en)-Gal4 driver at 18°C or 29°C. High levels of fusion proteins expression were confirmed by the AbdA (magenta) and Exd (with anti-HA, grey) immunostainings. Despite these high levels of protein expression, no BiFC can be visualized (upper images), highlighting the specificity of the methodology. [file 1741-7007-9-5-S5.pptx]

## Slide 1
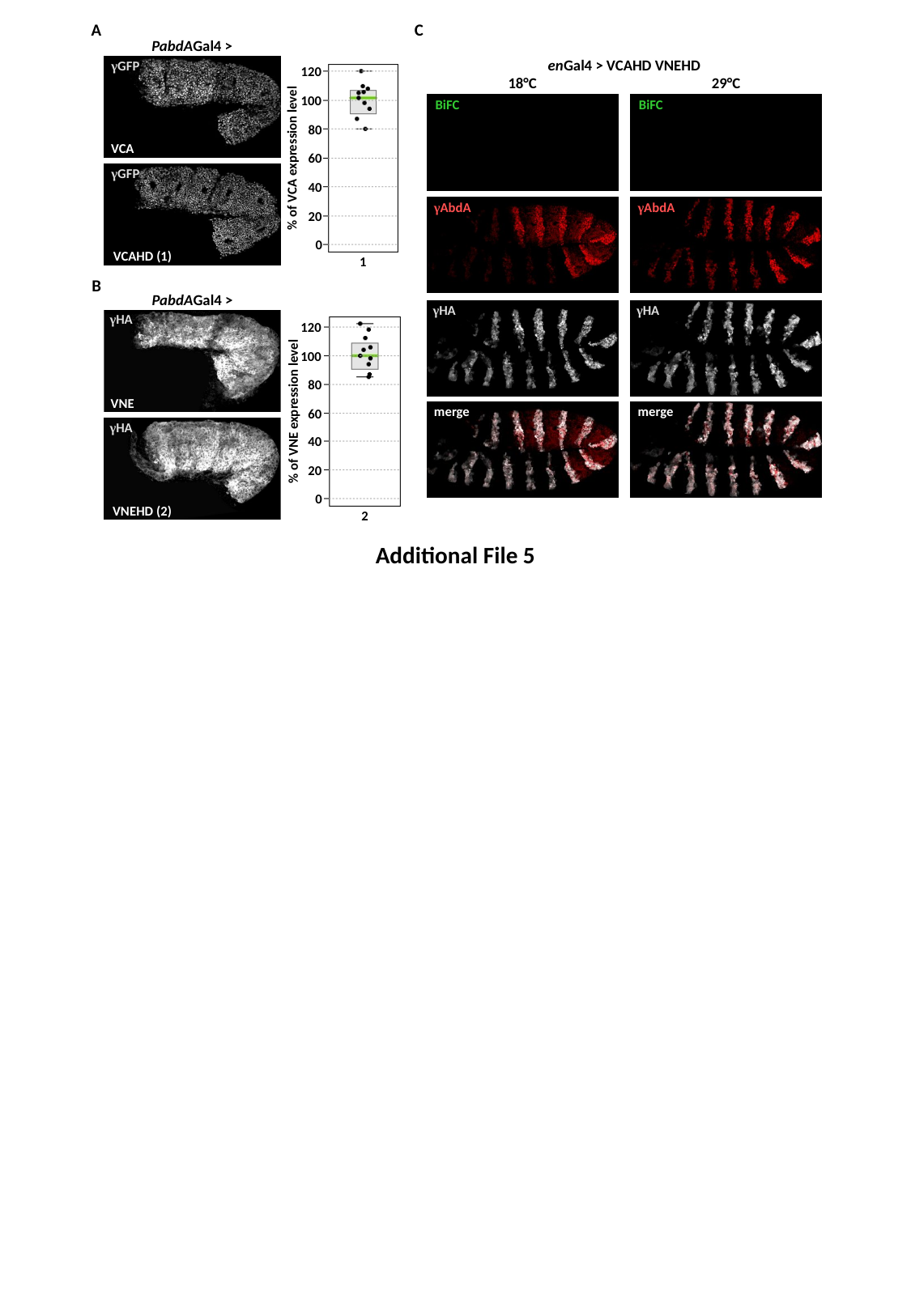

A
C
PabdAGal4 >
enGal4 > VCAHD VNEHD
γGFP
120
18°C
29°C
100
BiFC
BiFC
80
VCA
60
% of VCA expression level
γGFP
40
γAbdA
γAbdA
20
0
VCAHD (1)
1
B
PabdAGal4 >
γHA
γHA
γHA
120
100
80
VNE
merge
merge
% of VNE expression level
60
γHA
40
20
0
VNEHD (2)
2
Additional File 5
